# Book Reviews

**Published:** 1803-11-01

**Authors:** 


### CRITICAL ANALYSIS

OF THE

### RECENT PUBLICATIONS

ON THE

### DIFFERENT BRANCHES OF PHYSIC, SURGERY, AND MEDICAL PHILOSOPHY.

---

*Facts and Observations concerning the Prevention and Cure of Scarlet Fever, with some Remarks on the Origin of Acute Contagions in general.* By W. Blackburne, M. D. Octavo, pp. 170. London, 1803.

The principal object of this philanthropic writer, as far as relates to the scarlet fever, is to teach and recommend the means of preventing its spreading in schools and families.

" I conceive it, says he, to be my duty to communicate what information I have acquired, particularly with regard to the means of checking its progress; and, to enforce the necessity of attempting its total extinction, of which, as a specific contagion, it is completely capable. From these motives, the following result of my experience is made public.

" But it appears also, that the method of curing scarlet fever is not so consistent, or perfectly established, as the nature of *such* a disorder demands. So weighty and numerous are the exceptions to the general laws and precepts of medicine, when applied to individual cases, and so pernicious has been the indiscriminate adoption of precise rules in particular morbid affections, that the utmost caution is required in delivering them. Under this impression, a *General Outline only of the Treatment of Scarlet Fever* is here presented, ready to be filled up with deeper or lighter shades, according to the more acute perception and superior judgment of future observers.

" The history of its symptoms has been so faithfully executed by Dr. Withering, in his Treatise on Scarlet Fever; and by the late venerable Dr. Heberden, in his Commentaries, that it would be superfluous to detail them here.

Dr. Blackburne commences his work with a few general instructions and observations respecting the steps which should always be taken on the first appearance of this or any similar contagious disease in a public seminary or private family. As these rules appear to us judicious and important, we shall present our Readers with an abstract from them.

" As symptoms of indisposition do not immediately result from infection in a general way, no examination takes place with respect to the number of those who have been tainted when scarlet fever shows shows itself in a seminary; but as soon as the disease appears in one or more of the children, all are immediately dispersed. Those in whom the infection is latent, and to be afterwards produced, convey it to their respective families, where it seizes a less or a greater number of the individuals composing each family, particularly children and servants. By means of the latter, it is chiefly propagated through the circle of their acquaintance, and introduced into other families, and the disease is thus diffused through society without check or reserve. When the alarm is given in a numerous private family, a similar course is too often pursued; the members of it are usually sent away, inconsiderately, among their friends and acquaintance, and often to school, before it can be discovered whether the seeds of disease are not already sown in some of the fugitives. Several may even be infected from the same origin, in each of whom the disorder may appear at different intervals of time. But a very frequent and almost unsuspected source of infection, arises from the return of convalescents resuming the ordinary intercourse with the members of their family, *before* they have *ceased* to *disseminate contagion.* The precise time, in which the breath of a person who has passed through an infectious disease becomes incapable of conveying contagion, is by no means determined. The public are not sufficiently on their guard, with respect to this important fact. If the disease do not actually appear on the infected, they are apt to conclude, that a person in apparent good health cannot communicate disease. A very different conclusion will be drawn from the accounts I am about to recite in the sequel. And I feel it my duty most earnestly to recommend the utmost precaution and vigilance on these momentous points, universally, to the conductors of education and the heads of families.

" All masters and mistresses of boarding seminaries ought, for their own sake, to be provided with one or more separate apartments, in proportion to the size of their establishment, for the reception of invalids. These ought to be so contrived, that the communication between the sick rooms and the rest of the house may be easily, completely, and speedily cut off at any time. If the establishment be too contracted to admit of such appendages under the same roof, a lodging should always be kept in view in the neighbourhood; the owner of which, being instructed in the methods of preventing the spread of contagion, and suitably rewarded, might have apartments in readiness whenever occasion demanded. There are few institutions, however humble, which may not practise this simple mode of prevention; and none ought to be suffered, which do not fairly and fully comply with it. These regulations become daily more momentous, in the vicinity of London especially, as the great increase of the population, and the extension of its buildings, render public seminaries beyond the precincts of its impure atmosphere absolutely essential to the preservation of the rising generation.

" When

"When scarlet fever manifests itself in one subject, the firs precaution is to separate that subject from the rest without delay. The next very essential one is, to subdue unnecessary alarm and excessive fear; for as this disease is not communicable till the second or third day after its admission into any constitution, and previous or present fever almost always exists when it is admitted; and if it be made a general rule in all schools, to separate febrile patients of every description, according to Dr. Haygarth's recommendation; there will be time sufficient to apprise the childrens' friends of the accident, and to adopt that plan which shall most readily and most certainly extinguish the infection in the seminary, and also most effectually prevent its diffusion among society at large. In the performance of this great and indispensable duty, the parent and guardian must co-operate fully with the instructor; the interests, as well as the satisfaction of both, are deeply implicated in its due execution, and both will be amply rewarded for their mutual and best exertions by the most beneficial result.

"Where the scholars are numerous, and the extent and disposition of the premises admit of it, the best plan, both for parents, teachers, and the public, is *not* to disperse the school. Having ascertained and cut off the source of infection; having separated the originally tainted, as soon as they begin to sicken, and while they yet remain incapable of imparting disease; having disposed of them in proper apartments, and strictly enforced the rules of prevention; the evil may be crushed in its infancy. The extent and magnitude of the mischief will thus be accurately measured, and totally obviated. But if the accommodations of the establishment be too limited for the complete execution of this scheme, or parents be unwilling to commit their offspring to any other than their own inspection in the time of illness, it is a sacred duty imposed on them, not to admit even a suspected child, much less a diseased one, into family intercourse with themselves, their other children, or their servants. A separate apartment, with suitable furniture, where circumstances allow of such conveniences, ought to be always in readiness, or in a state to be made ready, on the shortest notice, for accidental sickness. Here a strict quarantine ought to be performed, whether the subject be suspecsed or convalescent, the period of which may be regulated, partly by what is already known on the subject, and finally determined by future observation and the result of aggregated facts, which hitherto have not been sought after with that zeal and diligence which their great importance demands. If the child be really infected, immediate separation, with the regimen essentially requisite to insure prevention, ought to be practised with the utmost strictness and precision. The rules of prevention being of difficult execution in large private families having numerous attendants, and the multiplication of the chances of spreading infection by removing young persons from school to their own home, are strong reasons why they should remain at school. One great mean of propagating contagion by these these removals is the custom of conveying infected persons in public vehicles, stage coaches, &c. This error, though committed daily, proceeds more from inattention and ignorance than any other motive; but when the impropriety and mischief of such a practice, and its highly pernicious consequences, are universally known and published, every individual ought conscientiously to abstain from these modes of imparting disease; and this resolution being once undeviatingly acted upon, public confidence would be fully established, all excessive alarms on the score of infection might safely subside, and even reasonable precaution might become less troublesome, and eventually be rendered unnecessary."

After Dr. B. has given a series of facts to serve as a foundation for his inferences, and made a number of valuable observations on the medical treatment of *Scarlatina Anginosa*, he proceeds "to inquire, by what variety of modes the infection of scarlatina may be introduced into the human body."

These he reduces to three, viz. 1st. Simple contact alone; 2d. Inoculation; 3d. Inhalation.

The first he thinks is very doubtful, and adduces sufficient arguments to establish the opinion. He even goes so far as to infer "that a degree of contact, which medical attention to, and nursing of the sick, renders necessary, is perfectly innocuous."

The second, or inoculation, very certainly communicates the specific disease when properly performed.

The third is that to which the Author appears desirous of calling the attention of the Profession in particular, and on which he founds his peculiar opinions respecting the origin of acute contagious diseases.

"Every species of contagion being suppressible by the temporary restriction of personal and intermediate communication, it follows, that the same restrictions are as practicable in all instances as in any single example. It cannot be denied, therefore, that the means of suspending the progress, or of locally annihilating all contagions, are as much in our power as the extinction of one.

"In the execution of a project so comprehensive as that which involves the suppression of every contagion, many obstacles will undoubtedly arise, which may, at first sight, appear insuperable. Similar objections have ever been opposed to every effort to meliorate the condition of man; but they have been most frequently overcome. Their opposition, on the present occasion, will not be more obstinate in an extended than in the limited plan, which is about to be put into execution. Great and numerous as the difficulties appear to be, they may all be included under the ignorance, the self-interest, and the indolence of human creatures. Ignorance on a subject of such high importance need not be suffered to remain long, where the means of information are so readily diffused as at the present æra, and the arm of the law can never be more properly called forth to lend its aid, than where selfishness, indolence, or insensibility to moral inducements are opposed to measures of undoubted undoubted public utility. No infringement on the genuine liberties of the subject is, on this occasion, proposed. In the instance of quarantine, men are obliged by law, to submit to restraints, which may transiently injure their interests, and interrupt, for a time, their social enjoyments; the more varied and extensive application of these laws is all that is required.

" A more forcible objection, however, and of a different character from the foregoing, remains to be obviated. Every species of infection must have had an origin. The same cause or causes which gave birth to a given contagious disease, in one or more individuals who first experienced it, will operate again under similar circumstances, and reproduce the same disease occasionally, notwithstanding the most successful attempts to extirpate it at any particular time. Allowing this objection its utmost force, the advantages will be infinitely great where prevention is instantly adopted, whenever such examples of original contagion occur, compared with the consequences which ensue, where no precautions are used. In support of this argument, a striking instance of the advanages of voluntary seclusion appears in its securing the health of the merchants of the Levant, in their factories, while the plague is destroying numbers all around them, no measures being taken by the people of the country to prevent its propagation.

" But the argument in favour of universal prevention becomes irrefragable, if the primary and principal sources of contagion can be discovered, and are then capable of being obviated or removed. With this view, a closer inspection into the origin of acute contagions in general may be instituted with advantage, which shall now be cursorily attempted, leaving to more able hands the completion of a research, than which none involves more extensively or more closely the dearest interests of our fellow creatures.

" The origin of contagious diseases has been commonly deemed so obscure, that any attempt to elucidate the subject would appear more curious than useful, more theoretical than practical, could it not be proved, that a more accurate acquaintance with infectious sources would most essentially promote that extensive plan for the extirpation of this class of diseases, which I am so anxious to recommend.

" We shall obtain the clearest view of our subject, by dividing all infectious diseases into the acute and the chronic. The first head alone shall occupy our attention at present, and under it are comprehended the *plague*, or *bubonary* fever; the *variolous*, *morbillous*, *scarlet*, and *yellow* fevers; and the *jail*, *hospital*, *putrid*, *low*, *nervous*, *malignant*, or *typhus* fever.

" In taking this enlarged survey of every description of infectious fever, I shall endeavour to establish the following positions on as solid a foundation as the difficult nature of the inquiry will permit, and apply them as closely as possible to the main point, which is the universal suppression of acute contagions.

" 1*st*. *Certain exhalations, or marsh miasmata, as they are usu-*

*ally termed, have the peculiar effect of inducing fever on human bodies, exposed, in certain conditions, to their influence.* From their denomination it is too commonly understood, that marshes are the only sources whence these exhalations arise. But they also proceed from moist earth, slime, mire, or mud, in a great variety of situations and climates, of inhabited as well as unfrequented and uncultivated tracts of country, in almost every quarter of the globe. They are more powerful, concentrated, and virulent in hot climates, and in hot seasons, than in temperate ones. And it appears that the types, or periodical evolutions of the fever which they excite, are chiefly governed by the degrees of concentration, which these exhalations possess. The type being more continued, and less intermittent or remittent, in proportion to the power of the exhalation; *animal febrile effluvia, cæteris paribus,* produce fever similar in type, in degree, and violence, to the strongest paludal exhala-" tions.

This first position constitutes the foundation of our author's theory, and therefore he illustrates and confirms it at considerable length; but as we think most of our readers will agree with him, we pass over his proofs.

His second position is, "*The effluvia from febrile animal bodies, and the exhalations from marshes, swamps, and mud, are gases of a peculiar composition, of which hydrogen, or the principle of humidity, forms an essential constituent part.* They do not appear to be the mere result of animal or vegetable putrefaction, although putrescent matter is most generally present, as requisite to their formation.

"Contagious pyrexial gas is not yet proved to be the result of simple deoxygenated human effluvia, unaided by, or uncompounded with, either febrile, or paludous, or limose gas.

"Although the precise composition of pyrexial gases, whether contagious, limose, or paludous, has not been discovered, yet, from a great number of facts, it is evident, that an aqueous constituent is essential to the composition of both. In the citations already extracted, to prove the effect of marsh miasmata, it has been amply demonstrated, that dampness, moisture, or humidity, is always an indispensable ingredient in the exhalations which induce fever. In the following passages the same truth is fully maintained. But another very important truth is also now, I believe, for the first time, brought to light, that, by depriving the pyrexial gases of their aqueous or hydrogenous principle, THEY ARE, FOR THE TIME, ANNIHILATED. THAT, UPON THIS SOLE PRINCIPLE, WE ARE ENABLED TO ACCOUNT FOR THE WELL KNOWN FACT, THAT EXTREME ADDITIONS OR ABSTRACTIONS OF CALORIC OR HEAT, ARREST THE PROGRESS, OR DESTROY THE EXISTENCE OF ALL EPIDEMIC AND CONTAGIOUS DISEASES.

"It will, in the next place, be requisite to adduce the evidence by which these circumstances, equal in magnitude and simplicity, are substantiated."

For

For most of this evidence we must refer our readers to the work itself; but the following passages we think too interesting to be omitted.

"There are no facts with which I am acquainted that prove accumulated human effluvia, from healthy bodies, uncompounded with limose or paludous gas, to be capable of generating fever. On the contrary, the following abridged account strongly militates against such an occurrence.

"A number of poor children, male and female, were removed in September, 1782, from Wimbledon to a large house in King-street, Golden-square. They continued in good health till the 8th of October. From this time a number of girls were seized in succession, at different intervals, with sickness, vomiting, sometimes purging, excruciating pain in the region of the stomach and in the back, which was soon followed by violent head-ach, delirium, and convulsions; they were in general costive. The symptoms were relieved by purgatives, but returned again each time with more violence.

'During the remission of the disease, they used to lie quiet 'during an hour, or even two hours, then suddenly to start up as 'before, screaming under the most afflictive torture, &c. They all 'agreed that, the fit approaching, their first sensation of pain was 'in the stomach; which having abated, the head, particularly the 'back part of it, was attacked in like manner; and it appeared, 'that a total perversion of the understanding very soon followed. '*None of them had any degree of fever, &c.* It was observable, that 'their paroxysms were always most severe after sleep. The dis'ease was shown only on certain of the girls, who slept in a par'ticular apartment. It contained ten beds, in which it was in'tended that eighteen girls, two in a bed, and a female servant 'singly, should sleep; but being a favourite room, on account of 'its warmth, it was generally crowded at night by a much greater 'number than its just complement. That as much space as possi'ble might be made for beds, the chimney had been stopped up 'with bricks, and it had been the custom of the servant at night 'to keep the door shut, and to close the window shutters, that as 'little fresh air as possible might have access. - - - From the time 'of the commencement of the illness, three candles and a lamp of 'oil had been used during the night; but they were hardly of any 'service, giving a glimmering light, and frequently almost extin'guished.'

"From the following statement it evidently appears, that the origin and cause of this disease consisted in an accumulation of human effluvia, uncombined with any other morbific gas, particularly pyrexial. For Sir George proceeds:

'It was likewise remarkable, that in a chamber adjoining, of the 'same dimensions, painted at the same time, in which eighteen 'girls slept in nine beds, (which chamber differed from the other 'only in having an open chimney, and not being so closely shut up 'during

'during the night,) none of the children had a symptom of the 'disease. Another striking fact was, that a female servant, who 'had passed one night only in attendance on the children in the 'chamber of the sick, was on the following morning attacked by 'the same pain, delirium, convulsions, &c.' Medical Transact. Vol. iii. p. 113.

The third position contains Dr. B's. Theory of the origin of acute contagions, viz.

"3dly. *The greatest number, if not all acute contagions, originate, in the first instance, from the exhalations or gases above specified: but they assume the property of propagating diseases similar to themselves, only under peculiar circumstances, which occasion their conversion from simple into contagious fever. The cause of this conversion is the exposure to accumulated* FEBRILIZED *animal effluvia.*

"The conversion of simple into contagious fevers is a fact established on the most satisfactory and undoubted testimony, part of which I shall now present to my reader. The same facts, which ascertain the above mentioned conversion, also demonstrate the cause to be that which I have just stated.

The evidence in support of this theory, is principally collected from the writings of those who have witnessed the springing up of contagious fevers in hot climates. The following passage will shew Dr. B's manner of applying his theory to the origin of particular diseases.

"That state of the human body, termed febrile, however various in its degrees, seems constantly associated with those contagious diseases which I have termed acute. But two circumstances are essential to formation of fever, a peculiar condition of the body, and its exposure to certain external circumstances, commonly styled remote and occasional causes. It will not be denied, that the condition of the body, at the time when it is attacked with fever, must greatly influence or vary the form of that disease which is about to become contagious, while the febrile actions, themselves, and the remote and proximate causes of those actions, may retain an uniform and identical character. The causes which determine one contagion to assume a pustulary form, as small-pox; another, a bubonary, as the plague; a third, eryspelatous, as scarlet fever, &c. are subjects of important inquiry, which have hitherto been very insufficiently investigated. I shall slightly touch upon them at present, for the sake of illustration, and of exciting discussion, rather than from any ability to form positive conclusions; but more especially, that by attempting to separate what is incidental from what is permanent, in the character of acute contagions, our views of these important diseases may be more clear and correct, their causes rendered less mysterious and obscure, the means of their prevention or their treatment more evident, more readily understood, and of more easy adoption. I shall select the small-pox, as my first example. From Mr. Denon's travels in Egypt, we find, in the following quotation, a very plausible origin origin of the small-pox, though it is not introduced by him with any view to such an application.

'The heat had become insupportable; the west wind oppressed 'us, caused bleedings of the nose, and painful eruptions, which 'covered, alternately, all parts of the body, dried and hardened 'the skin, and impeded perspiration. The rays of the sun, the 'principal, perhaps the sole cause of these evils, raised on every 'pore a pustule, similar to the small pox, which became intole-'rable, when, in lying down, it was necessary to rest on these 'points.' Denon, vol. II. p. 179. Aikin's translation.

"We have here, in my opinion, says Dr. B. the origin, outline, and external form of small-pox, exactly depicted, before its conversion into a contagious pest.

"A person under this pustular affection, being exposed to the causes which produce infectious fever in him, would become capable of communicating a disease named small-pox, to a second person receiving the effluvia of his body; and a third person being inoculated with matter taken from his pustules in a due state of maturity, would also be infected with the same disease. We see clearly, that the local affection or external form of a new disease may be easily acquired on exposure to new and peculiar external impressions; but it is extremely wonderful, that a new disease should, in the second instance of its existence, as well as in every future example, retain the precise form in which it originally appeared on the person first infected by it; such, however, is the fact. The small-pox has retained its original form, in passing through millions of victims, and under all the varieties of climate where it has appeared. If this origin of the small-pox be entitled to any degree of credibility or likelihood, its pustulary form is proved to be adventitious, while the attendant febrile actions will be found on comparison to bear the typhous stamp and character of other acute contagions, and most probably may claim a remote cause, similar or identical with theirs.

"Pursuing the same tract of investigation, we shall probably discover why the plague assumes a *bubonary* form. The excessive heat of the climate in Egypt, and its propinquity, in many parts, to sandy desarts, and the exposure of its inhabitants, and strangers or travellers, to the hot parching winds, which blow over them at certain seasons of the year, induce a peculiarly dry, irritable state of skin, and impeded perspiration. When perspiration is impeded, increased absorption takes place, and the lymphatic glands become distended and irritable. The cutis, deprived of its moisture, becomes parched, and susceptible of that inflammation which may be styled carbuncular, speedily terminating in mortification. *The intimate connection between the skin and lymphatic glands is well known, by various morbid affections, to exist in every climate.* An Egyptian, or any individual, being exposed to paludal gas, and attacked with fever, in this irritable state of skin and lymphatic glands, or when both are actually diseased, will be said to suffer the plague in his

 own own person; and if subjected to the circumstances which generally render fevers contagious, will communicate a disease of a specific form to a second individual receiving his effluvia."

---

### *Hey's Practical Observations on Surgery,*

( Concluded from the last Number. )

The fourth chapter of this valuable collection contains the history of several cases of a disease connected with the state of the blood-vessels, to which the author has given the name of Fungus Hæmatodes. The disease is a tumour coming on rather suddenly, generally, though not always, after some slight injury near the part affected, and increasing to an indefinite and often very enormous magnitude. The substance of the tumour is a soft fungus, somewhat resembling the brain in texture, and always affording a profuse hæmorrhage when broken or in any way divided. It appears to be partially organized, and uniformly vascular, but without any contractile power in the channels through which the blood oozes out. The seat is various, mostly contiguous to some muscles of the body, or their aponeurotic expansions; and when upon the breast, its fungous nature, and rapid growth, have often caused it to be mistaken for cancer. Mr. H. has found it always absolutely incurable and fatal except the suffering part be removed by amputation; and even in using this desperate remedy, the utmost care must be taken not to leave any portion of the disease, which would soon reduce the sound stump to the same morbid condition. The appearance which this disease assumes is better described by the author's own words.

"The tumour contained a very large quantity of a substance not much unlike coagulated blood; but more nearly resembling the medullary part of the brain, in its consistence and oily nature. It was of a variegated reddish colour, in some parts approaching to white; and, as blood issued from every part of it when bruised, I judged it to be uniformly organized. This mass was partly diffused through the circumjacent parts in innumerable pouches, to which it adhered; and was partly contained in a large sac of an aponeurotic texture. There was a great and universal effusion of blood from the internal surface of the sac, and from the pouches containing this morbid mass."

One of the cases here described, which terminated fatally, is illustrated by a very striking plate.

Dislocation is the subject of the next chapter; a species of accident, the treatment of which is sometimes attended with much difficulty, and not unfrequently resists the most powerful and the best directed means of reduction.

The following case shews much judgement in the operator, and the great command of strength possessed by one of the old methods of extension.

"September

" September 22d, 1775, a middle-aged man from Aldborough, near Boroughbridge, was admitted a patient of the General Infirmary, on account of a dislocation of the os humeri, at the shoulder, which had happened a month before his admission. The head of the bone lay behind the thick part of the pectoral muscle, and below the coracoid process of the scapula. Some attempts had been made to reduce the bone immediately after the accident, but without success.

" After he had lain in the warm bath about twenty minutes, the following methods were used to effect the reduction: After the arm was properly defended, straps, to which cords were affixed, were fastened by buckles upon the lower part, and he was drawn up gently from the ground by the help of pullies. Repeated trials by this method produced no sensible effect. We then used Freke's improved Ambi, and at one trial the bone suddenly advanced as if a reduction had taken place; but repeated efforts in this method had not the desired effect. We next made use of the methods recommended by Dr. Kirkland and Mr. White, placing a towel round the operator's neck, and holding back the inferior part of the scapula by means of a roller covered with cloths. Mr. Lucas and Mr. Jones afterwards tried to reduce the bone by the heel in the axilla, and Mr. Lucas perceived a noise during one effort, as if the bone had returned to its place. While the last method was in use, it occurred to me, that extension made in a direction parallel to that of the body was not likely to succeed, while the head of the bone lay so deeply sunk, and behind the pectoral muscle. I therefore advised that one person should extend the arm at right angles to the body, by the hold of the fore-arm, placing his foot against the side of the patient's thorax. In this way, the person making the extension would not only have a firm support, but would also be enabled to repress the lower part of the scapula by his heel placed against it. That during this extension, another person, lying by the side of the patient, should place his heel against the upper part of the os humeri, as near to its head as possible, and should push it in a direction parallel to that of the patient's body. By this method, the bone altered its situation with such a noise as is usually heard in reductions, and we concluded, that the head of the bone had re-entered the socket; but when the arm was brought close to the patient's side, we found that the head of the bone was still in the axilla. This appearance of success encouraged us, however, to repeat the operation, but the event was the same. We now imagined, that some portion of the capsular ligament might be folded so as to be intercepted between the head of the bone and the glenoid cavity, into which we judged the bone to have been twice brought. On this supposition, after making the reduction the third time, the os humeri was moved in various directions, sometimes upon its own axis, sometimes upwards and downwards, before we attempted to bring the arm towards the patient's side. Also, while the extension was continued, a flattened ball of tow was thrust up into the axilla by the heel, to prevent the head of the bone from retiring again into the axilla; the arm was then brought into contact with the patient's side, the extension being continued, though in a different direction, and the heel being gradually withdrawn as the arm approached the side. By these means the reduction was completed and confirmed. As the tendency to dislocation was so great, the arm was kept for a few days in contact with the side by a piece of girth web put round the arm and the body of the patient, who was dismissed cured."

Observations on different species of injuries of the larger joints, form the subjects of some of the succeeding chapters. They bear the same marks of accurate observation and good surgery.

The latter part of this volume consists of short observations on a variety of cases that are constantly occurring to the surgeon; some of them neither new nor of great moment; but the author invariably gives the result of his personal experience; and the perusal of this collection cannot fail to give a strong impression of the value of that experience. From the faithful testimony of Mr. Hey's case-book, which is here laid before the public, we conceive his practice to have been vigorous, decisive, judicious, and abounding with resources; and we do not hesitate to recommend this volume as worthy of a place in the library of every surgeon.

---

*The Charleston Medical Register, for the Year* 1802. *By* DAVID RAMSAY, M. D. Charleston. 12mo. pp. 22.

THE author of this pamphlet has been long so respectably known to the people of the United States as a writer and a practitioner of medicine, that to announce a publication from his pen will be sufficient to excite the attention of our readers. We are exceedingly pleased with the plan of this Register, which comprizes within a small compass a variety of important notices of subjects pertaining to medicine, and leaves us nothing to regret but the brevity with which some of them are treated. So highly prized are the abilities and experience of Dr. Ramsay, that whatever the pressure of his multiplied avocations compels him to curtail or to postpone in a performance of this kind, is felt by the public like the privation of a benefit to which a claim had been formally entered.

A view of the plan and objects of this Register will appear from the introduction prefixed to it, which we give in the words of the author:

" Medical facts, correctly stated and diligently compared together, reflect great light on the practice of physic. Conformable to this established principle, it must be obvious, that annual statements of the principal events connected with the health of the inhabitants, made by physicians in different places, would be particularly useful. The more extensively this was done, the better; but in the United States the advantages of such publications are enforced by peculiar considerations. In the old world, the attention of learned men has been been employed, for many centuries, in applying the general principles of medical science to the local peculiaritiss of each particular spot. Knowledge of this kind, in America, chiefly rests with individuals. To bring it within the reach of the community, requires the joint labours of practitioners in every part. If one physician, in each of the cities and towns of the United States, and several in the country parts of each state, were to favour the public with an annual account of the state of diseases, and of the circumstances connected with them, as far as their observations extended, there would, in time, be an accumulation of materials, from which we might obtain the following advantages :

" 1. More correct knowledge of the diseases of the United States.

" 2. A comparative view of the health and longevity of the inhabitants in different places.

" 3. Authentic evidences of all changes of the climate that took place ; and particularly of the effects produced on the health of the inhabitants from clearing and cultivating the soil, and from the different modes and articles of culture.

" 4. Persons labouring under any constitutional predisposition to particular diseases might select, with precision, a place of residence, least likely to call into action the particular predisposition under which they laboured. Such is the extent and variety of climates in the United States, that this might be done in almost every case, without changing the government or language to which persons proposing a change of residence were accustomed.

" 5. Physicians would be enabled to direct invalids to such a route in travelling as would best suit their particular habits and diseases. From the want of this local knowledge, improper advice is frequently given. The longitude and latitude of places afford no certain rule. Their influence, controled by a variety of local circumstances, is by no means uniform.

" The advantages of the proposed annual publications would not be confined to the medical department. The farmer and gardener, from an average of seasons, would be assisted in forming their opinion of the best time for their respective operations.

" The enterprising agriculturist, who wished to enrich his country with some new productions, would be informed when and where to make his experiments, by comparing the observations auxiliary to the practice of physic, with the usual habits of the particular commodity he wished to introduce.

" A facility might thus be given to the introduction of ginger, Japan sago, of the almond, allspice, caper, clove, cinnamon, camphor, nutmeg, red cotton trees, and several other valuable exotics. There are, doubtless, portions of the United States suitable to the culture of these articles ; but that suitableness is unknown to foreigners, and equally so to the owners of the soil. The same observation applies to the introduction of new animals, and of new branches of manufacture. Success, in both cases, must be materially influenced by the degree of heat and cold, and of the moisture and dryness of the atmosphere.

"The foreigner, who wished to remove to this part of the world, would also be enabled to determine where to locate himself, in a situation least variant from his trans-atlantic residence."

The following account of the diseases which chiefly prevailed in the course of the year, is presented to our readers:

"Charleston, in the year 1802, was afflicted with four epidemics; the small-pox, the measles, the influenza, and the yellow fever. There were cases of the small-pox in almost every month of the year. It proved fatal to four children, though inoculated for it by skilful physicians; and also to about twenty other persons, who took it in the natural way. These died under circumstances horrid to see, and painful to relate. Covered with confluent sores, they could neither stand, sit, nor lie, without exquisite pain. Their bodies and bed-clothes were stiffened with fœtid discharges from every part of their skin. The whole emitted a stench intolerable to bystanders. Humanity was put to the rack while it discharged the offices necessary for their support. In one case, an unfortunate negro (who caught the disease, remote from help, and unknown to his owner) was so far bereft of all power to help himself, that rats devoured a large portion of his tendo Achillis some days before he died. The recoveries from the small-pox were much more numerous than the deaths. Though in many cases, the subjects of the former suffered comparatively little, there were others who escaped with difficulty, and after a painful and distressing confinement.

"A considerable number, (supposed to be no less than twenty) took the small-pox in the natural way, after having been inoculated for it, and after medical practitioners had declared that they had the disease. One of these unhappy patients died, and others suffered more from it, in consequence of no pains having been taken to obviate a malady, from the attacks of which they supposed themselves to be free, till its advanced stages evinced the mistake.

"The general complexion of the diseases, for the first seven months of the year, was inflammatory. Pleurisies, acute rheumatisms, and complaints of the breast, were uncommonly frequent. From these precursors, some predicted a sickly summer, and a great prevalence of yellow fever; but they were agreeably disappointed. July, and the first seventeen days of August, were cool and healthy; there was only one day in both months in which the mercury in Fahrenheit's thermometer reached eighty-nine. The old inhabitants were generally free from diseases of every kind; and only two strangers died of the yellow fever before September. Fourteen of the last days of August, and the twenty-two first of September, were steadily warm; but not to so great a degree as in some former years. In only three of them (the 26th of August, and the 14th and 15th of September) did the mercury rise as high as 89. On the other hand, in only two of them (the 5th and 6th of September)

tember) did it fall below 80. In this warm season the yellow fever began to extend, but was less mortal than usual. More than half who were attacked by it recovered. The mode of treatment which seldomest failed, was depletion, followed by a mercurial salivation. There were a few, and only a few cases, where calomel produced its usual effects, in which the patient did not recover. Where the disease proved fatal, its superior excitement rendered this and every other medicine comparatively inert.

"A few strangers, though from northern latitudes, passed the summer (their first) in Charleston, without being attacked with the yellow fever. In three cases it proved fatal to persons who had resided in this city for eighteen months immediately preceding. No instance occurred of the death of any under twelve years of age from this disease.

"The eagerness of the people to receive their winter goods early in the season, induced men in trade to order matters so, that several vessels from foreign ports arrived in Charleston, with their unseasoned crews, in the months of August and September.

"To such the yellow fever was particularly inhospitable. To others arriving in the same season, Sullivan's Island afforded a safe retreat, till the danger was over. Exceptions to this have heretofore been very rare, and generally could be accounted for from some irregularity; but in the year 1802, five cases of the yellow fever (and two of them fatal) occurred in one house on that island, while the other inhabitants were generally healthy.

"No instance can be recollected, in which there was any ground to suppose that the yellow fever was either imported or had been contagious. No physician, nurse, nor other person exposed to contagion, from their intercourse with persons labouring under yellow fever, caught the disease. It was exclusively confined to strangers; and among them there was no evidence of its being communicated from one to another."

The introduction of the vaccine disease into Charleston is stated to have taken place in February, 1802, and to have succeeded in the most perfect manner. The author thinks that "nothing is now wanting to exterminate the small-pox but a general and simultaneous vaccination."

Among the medical improvements made in Charleston in the year 1802, Dr. R. mentions the establishment of a Dispensary, and the more active and energetic proceedings of the company incorporated in 1799, for the purpose of supplying the city with wholesome water from a pure and sufficiently distant source.

A regular series of meteorological observations is likewise included, which appears to have been made with great accuracy, and which affords a very satisfactory account of the temperature, &c. during that year.

The following interesting particulars of an attempt to rob the bank of South.Carolina, will, we are confident, appear to all our readers to be deserving of especial notice.

 "On

"On the night of the ninth of October, 1802, William Withers, a horse dealer from Kentucky, descended through a grate into one of the covered arch drains that pervades the streets of Charleston, and passed along the same, till he was opposite to the South-Carolina Bank. He then began operations to make a subterraneous passage across from the drain to the vaults in which the cash of the bank was deposited. In prosecuting this business, he passed ninety days and nights under ground, and in a prone posture. For the first twenty-two days after his descent, it was so uncommonly warm, as to be on an average nearly seventy-nine. For the last sixty-eight days the heat varied from seventy-four to thirty-three. In the first period, yellow fever, intermitting, and other fevers of warm seasons, were common among the inhabitants. In the last period, pleurisies, colds, and catarrhal complaints, were, in like manner, frequent: yet, all this time Withers enjoyed good health, with exceptions of a few slight head-achs and pains in his bones, which generally went off with perspiration in the course of his next repose. His situation in the drain was distressing; but it was tolerable; after passing through it, he was surrounded with earth. He had no blanket, nor covering of any kind, but his light ordinary apparel, which he had never put off. His usual time of sleeping was when he judged it to be day, from the noise he heard over his head. His signal for recommencing work was the receipt of provisions, dropt by his accomplices in the night, through a grate. He was sometimes exposed to serious danger from the springing of water; and his bed was earth, which was often damp. His food was mostly bread, butter, and cheese, and (with the exception of one bottle of wine) water was his only drink. Butter burning in a lamp afforded him light.

"Three days frequently passed without discharging the contents of his bowels.

"The enjoyment of so much health, for so long a time, under such circumstances, was, in part, probably owing to the following causes:

"1. A strong constitution, inured to hardships in every period of his life.

"2. That constitution suited to the air of Charleston, by a very recent seasoning. He had but just recovered from a severe fever when he entered the drain. Though relapses are not uncommon, yet a new and distinct fever scarcely ever attacks strangers in the same summer, in which they receive their first serious impressions from our climate.

"The effects of moisture must have been, in a great degree, parried by his labour, and the moisture itself moderated by the dry sandy nature of the soil through which he had to work, and by the absence of rain: for the first fifty days after his descent, the whole quantity of rain that fell did not amount to two-tenths of an inch; and in the last forty was only five inches eight-tenths; besides, sides, simple moisture, without heat or miasmata, is comparatively harmless.

"The absence of several of the exciting causes of diseases. The heat of well water and of the earth, a few feet below the surface, is generally the same in all countries as the medium heat on an average of the different seasons in these countries respectively. This, in Charleston, is sixty-five, or, at most, sixty-six on Fahrenheit's thermometer. Withers must have enjoyed a steady unvarying atmosphere of this temperature, while the inhabitants above ground were panting under a heat of eighty, or distressed with the cold of thirty-three, and subject to all the changes of an atmosphere, vibrating from one extreme to the other. That something in the air of Charleston, which is so destructive to strangers, in the summer and autumn, is too volatile to descend below the surface. Miners and colliers in all countries are generally healthy.

"The experiment is not recommended; but it is probable that a subterranean residence might be so constructed as to afford security against our local diseases.

"The great excitement of Withers' mind, from the prospect of accumulating wealth, must have counteracted the effects that otherwise would naturally have resulted from his situation. The energies of human nature, when in pursuit of a great object, (especially if invigorated with the hope of obtaining it) are beyond all calculation. The weakly wife, and the tender mother, will undergo watchings and fatigues in nursing the object of their affection, far beyond the power of human nature to bear, when in a state of indifference. The high toned state of Withers' mind must have had a decided influence in preserving his health: It is much to be regretted that it was not excited by worthy objects.'"

We hope the author will be induced, by the considerations which he urges with so much force and good sense, annually hereafter to continue his Register; and that other physicians, in different parts of the United States, will become emulous to follow his good example.

---

*A Treatise on the Cow-Pox, containing the History of Vaccine Inoculation, and an Account of the various Publications which have appeared on that Subject, in Great Britain, and other Parts of the World.* By John Ring. Part II. 8vo. p p. 567. London, 1803.

This correct, indefatigable, and successful promoter of Vaccination, has at length completed his elaborate history of the subject, down to the present time. His industry and talents for such a task are sufficiently known to those who are acquainted with him, or have read his first volume, to which this is in no respect inferior. As the Medical Journal contains a very full history of the cow-pox, at least all the more important facts, our readers will not expect us to give extracts from the present work. This

2d Part

2d Part is accompanied with a coloured plate, which gives a very excellent representation of the progress of the vaccine vesicule through all its stages, and there is added a copious Index to both Parts.

